# Psychiatric standardized claim ratios by rurality and deprivation in Japan: A nationwide ecological study with contextual suicide mortality

**DOI:** 10.1002/pcn5.70392

**Published:** 2026-08-10

**Authors:** Masahide Koda, Shuhei Nomura, Makoto Kaneko, Nahoko Harada

**Affiliations:** ^1^ Division of Epidemiology, School of Public Health Tohoku University Graduate School of Medicine Sendai Miyagi Japan; ^2^ Global Health Policy Lab, International Research Institute of Disaster Science (IRIDeS) Tohoku University Sendai Miyagi Japan; ^3^ Global Health Policy Lab, Graduate School of Medicine Tohoku University Sendai Miyagi Japan; ^4^ Department of Health Policy and Management, School of Medicine Keio University Tokyo Japan; ^5^ Department of General Medicine Kitasato University School of Medicine Sagamihara Kanagawa Japan; ^6^ Sagamihara Endowed Chair in Comprehensive Community Medicine Kitasato University School of Medicine Sagamihara Kanagawa Japan; ^7^ Faculty of Interdisciplinary Science and Engineering in Health Systems Okayama University Okayama Japan

**Keywords:** Area Deprivation Index, community mental health services, rural health services, standardized claim ratio, suicide

## Abstract

**Aim:**

We examined whether rurality was associated with psychiatric standardized claim ratios computed using medical‐institution location, and how these patterns related to area deprivation and residence‐based suicide mortality in Japan.

**Methods:**

We conducted a nationwide ecological study of 335 secondary medical areas. Rurality, measured with the Rurality Index for Japan, and a census‐based Area Deprivation Index were population‐weighted from municipalities. Psychiatric specialty therapy claims (April 2023 to March 2024) from Japan's national health‐insurance claims open data were expressed as standardized claim ratios: observed claims billed by institutions in each area divided by national age–sex expected claims. Suicide deaths (April 2022 to March 2025) were expressed as age–sex standardized mortality ratios. Ecological rate‐ratio trends were estimated with quasi‐Poisson models.

**Results:**

Outpatient claim ratios were lower in rural than urban areas but non‐monotonic (most urban, 108.4; fourth quintile, 76.9; and most rural, 81.3). Inpatient ratios peaked in the second quintile, and suicide mortality rose with rurality. Per 10‐point higher rurality score, outpatient volume was lower (rate ratio, 0.939; 95% confidence interval, 0.921–0.956) and remained lower after including deprivation (0.919; 0.896–0.942); the rurality–suicide association attenuated. Nearest‐three‐area pooling raised the most rural median outpatient ratio from 62.2 to 101.1.

**Conclusion:**

More rural areas had lower outpatient psychiatric claim volume billed by local institutions relative to national age–sex benchmarks, but this contrast narrowed after nearest‐area pooling, consistent with cross‐boundary care. These institution–location ratios should prompt local review with residence‐based and capacity data, not be read as evidence of under‐provision.

## INTRODUCTION

Japan provides an informative setting for social psychiatry because universal health insurance, a historically large psychiatric inpatient sector, wide regional variation in beds and services, and policies to strengthen community‐based mental health care coexist.^1^, ^2^, ^3^ National policy promotes community‐based integrated care for people with mental disorders and support for community living beyond inpatient‐centered care.^4^ Japan's annual 630 survey provides essential facility‐oriented snapshots of psychiatric facilities and patients.^5^ A central question is therefore whether the outpatient and inpatient psychiatric claims billed by institutions located in an area differ systematically between rural and urban settings.

Rurality is relevant because depopulation, population aging, travel distance, remote islands, and weather‐related access may shape where psychiatric services are delivered and how care is organized. The psychiatrist workforce in Japan may become increasingly strained under demographic change.^6^ At the same time, rurality may overlap with area‐level socioeconomic deprivation, which can influence health through household composition, employment, housing, social isolation, and material resources. The Japanese census‐based Area Deprivation Index (ADI) offers one way to distinguish deprivation from geographic remoteness.^7^, ^8^ Routinely collected service‐use data also require careful interpretation: claims tabulated by institution location indicate where services are billed and may reflect provider location, coding, service intensity, institutional history, and cross‐boundary care rather than the care received by residents.

Mental disorders contribute substantially to population health burden, and treatment gaps are well documented internationally and in Japan.^9^, ^10^, ^11^, ^12^, ^13^, ^14^ The World Health Organization calls for transforming mental health systems toward community‐based care, increasing the need for routine, population‐benchmarked service indicators.^15^ Japan, combining a historically large inpatient sector with an explicit community‐transition policy, offers internationally relevant lessons on how provider‐location claims can, and cannot, serve as planning indicators. Recent Japanese studies have used the National Database of Health Insurance Claims and Specific Health Checkups (NDB) Open Data to describe psychiatric day care, psychiatric occupational therapy, and child and adolescent outpatient psychiatric treatment,^16^, ^17^, ^18^ and ecological studies have examined rurality, area deprivation, and mortality or suicide in Japan.^19^, ^20^, ^21^, ^22^ However, these lines of evidence have largely been pursued separately. Evidence that jointly considers rurality, locally billed psychiatric claim volume, area deprivation, and residence‐based suicide mortality within the same policy‐relevant geographic units remains limited. This matters for psychiatry because the same area may simultaneously show lower locally billed outpatient claim volume, a different inpatient profile, greater deprivation, and higher suicide mortality, patterns that single‐dimension analyses cannot disentangle.

Existing regional indicators, however, mainly describe stocks: psychiatrist density and bed availability describe capacity, and the 630 survey describes facilities and patients at a reference date.^5^ None of these shows whether institutions located in a planning unit generate psychiatric specialty therapy claims above or below the volume expected from national age–sex claim rates; a planner can know how many psychiatrists, beds, or facilities an area has without knowing whether locally billed claim volume matches the age–sex benchmark. The standardized claim ratio (SCR) examined here adds this annual claims‐flow signal to be triangulated with residence‐based access, capacity, and need data rather than used alone.

Two data resources make such a joint analysis feasible at the level of the secondary medical area, the unit used for regional health planning. The Rurality Index for Japan (RIJ) was developed and validated for health care research using factors relevant to the Japanese context, including population density, distance to hospital, remote islands, and weather‐related access,^23^ and updated RIJ variants were developed using a recent census and a modified definition of remote islands.^24^ Japan's NDB Open Data provides nationwide aggregated psychiatric specialty therapy counts by secondary medical area and medical‐institution location.^25^, ^26^

In this nationwide ecological study, we examined whether rurality was associated with outpatient and inpatient psychiatric specialty SCRs computed using institution location, and whether these patterns co‐occurred with area deprivation and residence‐based suicide mortality. Using an established Japanese planning metric, we aimed to clarify whether rurality, deprivation, institution–location claim volume, and residence‐based suicide mortality represent overlapping but distinct dimensions for regional psychiatric service review. We also assessed whether these dimensions could serve as candidate indicators for prioritizing areas that warrant local assessment of patient flow, workforce, travel, service capacity, and care pathways.

## METHODS

### Study design and reporting

We conducted an ecological cross‐sectional study of Japanese secondary medical areas using aggregate statistics, with no individual‐level patient information. The manuscript follows the Strengthening the Reporting of Observational Studies in Epidemiology (STROBE) and the REporting of studies Conducted using Observational Routinely‐collected health Data (RECORD) guidelines.^27^, ^28^

### Geographic unit

The analytic unit was the secondary medical area. In Japan's health planning system, each secondary medical area comprises multiple municipalities and is designed so that routine hospital care, including emergency medicine, can be completed within its boundaries. We mapped municipalities to secondary medical areas using National Land Numerical Information medical area data^29^ yielding 335 secondary medical areas.

### Exposure: Rurality Index for Japan

The exposure was RIJ aggregated to secondary medical areas by population weighting. RIJ was categorized into quintiles, with Q1 the most urban and Q5 the most rural; each quintile contained 67 secondary medical areas (Table 1). Continuous RIJ was also modeled per 10‐point increase. We used municipality‐level RIJ 2024 scores from the updated RIJ family, in which higher scores indicate greater rurality.^24^ Specifically, we used the mRIJ‐direct variant, constructed from population density, straight‐line distance to the nearest secondary or tertiary emergency hospital, modified island coding, and special heavy‐snowfall area status. Municipality‐level mRIJ‐direct scores ranged from 1 to 100. Population‐weighted secondary‐medical‐area scores ranged from 2.876 to 97.985; the RIJ 2024 variant specification and quintile cut‐points are provided in Table S2.

**Table 1:** Selected Rurality Index for Japan (RIJ) quintile‐level core psychiatric service, suicide, and deprivation indicators.

Indicator	Q1 most urban	Q2	Q3	Q4	Q5 most rural
SMAs, *n*	67	67	67	67	67
Population aged 20+, *n*	56,811,647	22,176,057	13,330,834	8,163,496	4,426,035
RIJ, median	23.028	42.727	53.354	61.000	77.478
ADI rank, median	16	36	52	71	76
Outpatient SCR, group‐level	108.4	95.7	90.6	76.9	81.3
Outpatient SCR, area‐level median (IQR)	93.9 (83.2–115.1)	95.4 (77.9–114.9)	85.4 (68.5–105.9)	72.9 (60.9–88.9)	62.2 (46.1–91.3)
Areas with outpatient SCR < 100, *n*/67	40/67	40/67	47/67	53/67	51/67
Outpatient observed/expected claims, millions	48.48/44.71	16.47/17.21	9.28/10.25	4.77/6.21	2.71/3.33
Inpatient SCR, group‐level	83.6	127.8	106.7	111.5	108.8
Inpatient SCR, area‐level median (IQR)	75.3 (49.8–123.3)	116.8 (75.6–189.5)	92.9 (51.6–152.6)	112.0 (72.3–157.1)	99.8 (39.3–138.3)
Areas with inpatient SCR > 100, *n*/67	25/67	42/67	27/67	41/67	33/67
Inpatient observed/expected claims, millions	19.69/23.54	12.78/9.99	6.62/6.21	4.45/3.99	2.46/2.26
Suicide SMR, group‐level	0.961	0.999	1.008	1.124	1.257
Suicide SMR, area‐level median (IQR)	0.948 (0.887–1.023)	1.007 (0.912–1.106)	1.030 (0.902–1.144)	1.142 (1.005–1.286)	1.224 (1.034–1.443)
Areas with suicide SMR > 1.0, *n*/67	20/67	36/67	39/67	52/67	55/67
Annualized suicide rate per 100,000	16.05	16.68	16.83	18.76	20.98
Low outpatient SCR + high inpatient SCR, *n*	13	20	14	29	20
Low outpatient SCR + high inpatient SCR + high suicide SMR, *n*	4	9	9	23	16

*Note*: Group‐level SCRs are calculated as 100 × summed observed claims divided by summed age–sex expected claims within each RIJ quintile. Area‐level medians and IQRs summarize the distribution of SCRs across the 67 secondary medical areas in each quintile, giving equal weight to each area; group‐level and area‐level summaries are distinct. Observed/expected claim totals in this table are shown in millions.

Abbreviations: ADI, Area Deprivation Index; IQR, interquartile range; SCR, standardized claim ratio; SMA, secondary medical area; SMR, standardized mortality ratio.

### Area Deprivation Index

Area‐level socioeconomic deprivation was measured with the Japanese census‐based ADI, a composite of eight census indicators covering household composition, housing tenure, labor‐force status, and occupational structure.^7^, ^8^ Higher A DI indicates greater deprivation. Municipality‐level ADI values based on the 2020 Census were aggregated to secondary medical areas as population‐weighted means and expressed as percentile ranks from 1 to 100 across the 335 areas. ADI was based on the 2020 Census, the most recent census for which the component tables required to construct the index were available.^30^, ^31^

### Psychiatric service outcomes

The primary service outcome was the NDB Open Data Category I psychiatric specialty therapy outpatient claim count, excluding the reimbursement add‐on, tabulated by secondary medical area for April 2023 to March 2024.25 The NDB Open Data tables separate this item group into claims with and without a supplementary add‐on; the primary outcome used claims excluding the add‐on to preserve a stable base service category. The full set of official fee‐schedule item names, reimbursement category codes, service codes, points, source workbooks, and analysis roles is listed in Table S1; examples include inpatient psychiatric therapy (I001) and outpatient/home‐care psychiatric therapy (I002). Because the NDB Open Data documentation states that prefecture and secondary‐medical‐area tabulations for medical claims are based on medical‐institution location, we interpreted these outcomes as claim volume billed by institutions located in the area, not as the care received by residents.

Sensitivity outcomes were outpatient claim counts including the add‐on and outpatient patient counts with and without the add‐on. Inpatient claim counts excluding the add‐on were analyzed as the primary contrast indicator of service mix. Patient‐count analyses served as robustness checks because national age–sex patient‐count rates were unavailable.

### Suicide mortality outcome

As a contextual residence‐based outcome, we used the Ministry of Health, Labour and Welfare Basic Data on Suicide in Communities, A7 municipal tables by suicide date and residence.^32^ We pooled 36 months from April 2022 to March 2025, centered on the NDB 10th Open Data service period. Municipal suicide counts were aggregated to secondary medical areas using the medical‐area correspondence table. Municipalities without listed deaths were assigned zero counts before aggregation.

We used indirect age–sex standardization. National age–sex suicide rates for the same 36‐month period were applied to each area's age–sex population from Basic Resident Register data.^33^ The suicide standardized mortality ratio (SMR) was observed deaths divided by expected deaths. Suicide statistics were used as a contextual population outcome, not as a direct outcome of psychiatric service volume.

### Population denominators and facility measures

Municipality‐level Basic Resident Register population by 5‐year age group and sex was used to construct secondary‐medical‐area populations.^33^ The adult population denominator was used for descriptive summaries and adult‐population comparator sensitivity analyses, whereas SCR expected counts used the national age–sex strata corresponding to the NDB age–sex claim tables. National age–sex denominators for SCR claim rates were taken from the 2024 Basic Resident Register national male and female rows. The 2025 Census preliminary release did not yet provide the age–sex strata required for this calculation.^30^, ^31^ Psychiatric facility measures were derived from Medical Information Net Open Data as of December 1, 2024.^34^ Facility counts were location proxies, not workforce capacity measures. Age ranges and denominators for each indicator are summarized in Table S5.

### Standardized claim ratio for psychiatric service volume

The primary service‐volume indicator was a psychiatric SCR computed using institution location, following the observed‐to‐expected logic of Japan's NDB‐based SCR tools for medical care planning35^35^ and of indirect standardization and small‐area variation analyses.^36^, ^37^, ^38^ For secondary medical area *i*, observed service volume was the NDB claim count billed by medical institutions located in that area. Expected counts were the sum across age–sex strata of area population multiplied by the corresponding national psychiatric specialty therapy claim rate; the national rate was national NDB age–sex claim counts divided by national 2024 Basic Resident Register age–sex population. SCR was observed claims divided by expected claims, multiplied by 100, so that 100 represents the national age–sex claim‐rate benchmark. The source masking rule, observed masked‐cell counts, expected‐count rescaling constants, and formula are shown in Table S3.

Because secondary‐medical‐area NDB tabulations are based on institution location, this SCR is an institution–location claim‐volume indicator. An SCR below 100 indicates fewer locally billed claims than the national age–sex benchmark; it does not indicate the care received by residents, because low SCR may reflect cross‐boundary care, coding, service intensity, or institutional history. In the NDB Open Data medical‐claim tables used here, source cells below the minimum aggregation unit (<10) are suppressed, and complementary suppression may be applied to prevent back‐calculation. Suppressed source cells were set to zero before aggregation.

### Statistical analysis

We summarized SCR by RIJ quintile using group‐level observed and expected totals, the area‐level median and interquartile range (IQR) of SCR, and the number of areas beyond the a priori cut‐points defined below, and estimated Spearman correlations between continuous RIJ and SCR. For model‐based summaries, we fitted Poisson log‐link models with observed counts as the outcome and log age–sex expected counts as an offset. Because psychiatric claim counts are high‐volume administrative counts with substantial between‐area heterogeneity, quasi‐Poisson standard errors were used to summarize ecological rate‐ratio trends relative to expected counts; the models were not intended as individual‐level, causal, predictive, or mechanistic utilization models. RIJ was modeled per 10‐point increase and, in categorical descriptions, by quintile with Q1 as reference. For the overlap analysis, descriptive cut‐points for low outpatient claim volume, high inpatient claim volume, and high suicide mortality were defined a priori as outpatient SCR below 100, inpatient SCR above 100, and suicide SMR above 1.0.

To assess sensitivity to threshold placement, we repeated the overlap counts after varying one cut‐point at a time (outpatient and inpatient SCR thresholds of 90–110 in steps of 5; suicide SMR thresholds of 0.90–1.10 in steps of 0.05) and after evaluating all cut‐point combinations. We counted areas within near‐threshold bands (outpatient and inpatient SCR 95–105; suicide SMR 0.95–1.05) as a descriptive measure of areas that could change category with small threshold shifts.

For suicide mortality, we summarized group‐level SMR and the annualized indirect age–sex standardized suicide mortality rate by RIJ quintile, and estimated Spearman correlations between suicide SMR, RIJ, ADI, psychiatric SCR, and facility‐density measures. Socioeconomic sensitivity models added secondary‐medical‐area ADI rank per 10 points to the quasi‐Poisson models for outpatient claim volume, inpatient claim volume, and suicide deaths; additional models used ADI rank *z* scores and raw ADI *z* scores (Table 2). Facility‐density correlations are reported in Table S4.

**Table 2:** Descriptive quasi‐Poisson offset model summaries.

Outcome	Model	Term	Rate ratio (95% CI)	Dispersion phi
Outpatient psychiatric specialty therapy claims	RIJ only	RIJ per 10 points	0.939 (0.921–0.956)	25,945.2
Outpatient psychiatric specialty therapy claims	RIJ + ADI rank	RIJ per 10 points	0.919 (0.896–0.942)	25,653.2
Outpatient psychiatric specialty therapy claims	RIJ + ADI rank	ADI rank per 10 points	1.026 (1.006–1.047)	25,653.2
Inpatient psychiatric specialty therapy claims	RIJ only	RIJ per 10 points	1.091 (1.056–1.127)	53,582.5
Inpatient psychiatric specialty therapy claims	RIJ + ADI rank	RIJ per 10 points	1.019 (0.977–1.064)	48,070.5
Inpatient psychiatric specialty therapy claims	RIJ + ADI rank	ADI rank per 10 points	1.082 (1.047–1.118)	48,070.5
Suicide deaths	RIJ only	RIJ per 10 points	1.030 (1.023–1.037)	3.1
Suicide deaths	RIJ + ADI rank	RIJ per 10 points	1.007 (0.998–1.016)	2.6
Suicide deaths	RIJ + ADI rank	ADI rank per 10 points	1.028 (1.021–1.035)	2.6

*Note*: Rate ratios are descriptive ecological trend summaries relative to age–sex expected counts. Outcomes are outpatient and inpatient psychiatric specialty therapy claim counts and suicide deaths.

Abbreviations: ADI, Area Deprivation Index; CI, confidence interval; RIJ, Rurality Index for Japan.

### Sensitivity analyses

We performed sensitivity analyses using alternative NDB outcomes; excluding the temporary COVID‐19 reimbursement code (I999) from the primary outpatient outcome; imputing suppressed source cells at the midpoint value of 5 instead of 0, with national age–sex claim rates, expected counts, and rescaling factors recalculated; excluding secondary medical areas with adult population below 50,000 or 100,000; excluding island secondary medical areas; aggregating to prefectures; and pooling each secondary medical area with its three geographically nearest areas identified using approximate geographic representatives from the secondary‐medical‐area boundary polygons. Pooling three nearest areas was a pragmatic boundary‐sensitivity choice rather than a prespecified or literature‐derived catchment definition; we therefore repeated the pooling with two and four nearest areas and after excluding island focal areas (Table S9). We also calculated prefecture‐clustered standard errors for the RIJ + ADI models. As a spatial diagnostic, we used the nearest‐three‐area structure to calculate Moran's *I* for outpatient SCR, inpatient SCR, suicide SMR, and selected RIJ + ADI Pearson residuals.

### Ethics

This study used aggregate administrative statistics without individual‐level identifiable information. Formal ethics review and consent were not required for this aggregate‐data analysis under the applicable institutional policy.

## RESULTS

### Data assembly

The analytic service‐volume dataset comprised 335 secondary medical areas and 104,908,069 residents aged 20 years or older. Nationally, the primary outpatient psychiatric specialty therapy outcome (excluding the add‐on) contained 81,717,926 observed claims, and the inpatient contrast outcome contained 45,997,763. ADI values were assigned to all 1890 municipalities in the analysis. The suicide analysis used 62,550 mappable, age‐classified suicide deaths over 36 months.

### Rurality, claim volume, deprivation, and suicide: Core indicators

Outpatient psychiatric specialty therapy claim volume was higher than age–sex expected in the most urban quintile and lower than expected in rural quintiles: group‐level outpatient SCR was 108.4 in Q1, 95.7 in Q2, 90.6 in Q3, 76.9 in Q4, and 81.3 in Q5. Area‐level median outpatient SCR was 93.9 (IQR 83.2–115.1) in Q1 and 62.2 (IQR 46.1–91.3) in Q5; areas with outpatient SCR below 100 were 40/67 in Q1 and 51/67 in Q5, peaking at 53/67 in Q4. Group‐level inpatient SCR was 83.6 in Q1, 127.8 in Q2, 106.7 in Q3, 111.5 in Q4, and 108.8 in Q5. Area‐level median inpatient SCR was 75.3 (IQR 49.8–123.3) in Q1 and 99.8 (IQR 39.3–138.3) in Q5; areas with inpatient SCR above 100 ranged from 25/67 in Q1 to 42/67 in Q2 and 33/67 in Q5.

Areas combining low outpatient SCR, high inpatient SCR, and high suicide SMR were more common in Q4 and Q5 than in Q1–Q3. Using the a priori cut‐points (outpatient SCR below 100, inpatient SCR above 100, and suicide SMR above 1.0), the three‐condition counts were 4, 9, 9, 23, and 16 across Q1–Q5; the two‐condition counts for low outpatient and high inpatient SCR, regardless of suicide SMR, were 13, 20, 14, 29, and 20.

In one‐at‐a‐time threshold‐sensitivity analyses, Q4 and Q5 accounted for 54.2%–77.5% of areas meeting all three overlap conditions, and for 48.2%–58.3% of areas with low outpatient and high inpatient SCR; across the full threshold grid, the corresponding Q4 + Q5 share was 49.3%–81.6% for the three‐condition definition and 46.3%–58.3% for the two‐condition definition (Table S8). Near‐threshold bands contained 31 areas for outpatient SCR 95–105, 32 for inpatient SCR 95–105, and 84 for suicide SMR 0.95–1.05.

### Descriptive rate‐ratio models and ADI adjustment

Continuous RIJ correlated negatively with outpatient SCR (Spearman rho −0.380) and positively with suicide SMR (rho 0.476), while its correlation with inpatient SCR was near zero (rho 0.008). ADI rank correlated with RIJ (rho 0.680), suicide SMR (rho 0.543), and inpatient SCR (rho 0.207), and inversely with outpatient SCR (rho −0.205). Psychiatric facility density per 100,000 correlated positively with outpatient SCR (rho 0.316) and inpatient SCR (rho 0.200), and was near zero with RIJ (rho −0.028) and suicide SMR (rho 0.022); clinic‐ and hospital‐specific facility‐density correlations are presented in Table S4.

ADI rank was essentially uncorrelated with total psychiatric facility density (rho 0.004), inversely correlated with psychiatric clinic density (rho −0.338), and positively correlated with psychiatric hospital density (rho 0.458) (Table S4).

In descriptive offset models, each 10‐point higher RIJ score was associated with lower outpatient claim volume relative to age–sex expected counts (rate ratio 0.939, 95% confidence interval 0.921–0.956), and this association persisted after adding ADI rank (rate ratio 0.919, 95% confidence interval 0.896–0.942). For inpatient claims, the RIJ‐only rate ratio was 1.091 (95% confidence interval 1.056–1.127), which attenuated to 1.019 (95% confidence interval 0.977–1.064) after adding ADI rank. In the RIJ + ADI models, each 10‐point higher ADI rank was associated with higher outpatient claims (rate ratio 1.026, 95% confidence interval 1.006–1.047), higher inpatient claims (rate ratio 1.082, 95% confidence interval 1.047–1.118), and higher suicide deaths (rate ratio 1.028, 95% confidence interval 1.021–1.035). Quintile‐categorical models showed consistent patterns; for example, the Q5 versus Q1 outpatient rate ratio was 0.750 (95% confidence interval 0.613–0.918) in the RIJ‐only model and 0.701 (95% confidence interval 0.560–0.879) after adding ADI rank (Table S6).

### Sensitivity and spatial diagnostics

The lower rural outpatient pattern was generally observed across alternative outcomes, standardization choices, population restrictions, island exclusion, aggregation, clustered standard errors, and spatial diagnostics, but was substantially attenuated under nearest‐three‐area pooling (Table 3). Excluding areas with fewer than 50,000 adults produced a Q5 outpatient SCR of 83.9; excluding areas with fewer than 100,000 adults produced 94.3, although only 12 Q5 areas remained. Excluding island areas produced a Q5 outpatient SCR of 83.5. Nearest‐area pooling raised the Q5 median outpatient SCR from 62.2 to 98.1, 101.1, and 99.8 when each area was pooled with its two, three, and four geographically nearest areas; excluding island focal areas, the nearest‐three‐area pooled median was 96.4 (Table S9). The RIJ‐outpatient estimate in the RIJ + ADI model with prefecture‐clustered standard errors was 0.919 (95% CI 0.894–0.945). Nearest‐three‐area Moran's *I* values were 0.084 for outpatient SCR, 0.406 for inpatient SCR, 0.238 for suicide SMR, and 0.204 for RIJ + ADI suicide‐model residuals. Excluding the temporary COVID‐19 code, which accounted for 0.038% of outpatient source claims, left the outpatient SCRs and rate ratio unchanged, and imputing suppressed cells at the midpoint changed the Q5 outpatient group‐level SCR from 81.3 to 81.5 and the Q5 median from 62.2 to 62.4 (Table S7).

**Table 3:** Robustness and diagnostic summary.

Check	Result	Reported detail
Earlier adult‐population comparator	Q1 109.5; Q5 78.6	Adult‐population comparator
Primary age–sex SCR	Q1 108.4; Q5 81.3	Primary claim‐count SCR
Alternative outpatient outcomes	With‐add‐on Q1 108.2; Q5 80.3; patient‐count Q5 73.1–74.0	Add‐on and patient‐count diagnostics
Exclude SMAs with adult population <50,000	Q5 83.9; *n* = 32 Q5 SMAs	Population restriction
Exclude SMAs with adult population <100,000	Q5 94.3; *n* = 12 Q5 SMAs	Population restriction
Exclude explicit island SMAs	Q5 83.5; *n* = 54 Q5 SMAs	Island restriction
Nearest‐three‐SMA pooled median	Q5 median 101.1; 98.1 with two and 99.8 with four nearest areas	Nearest‐area pooled
Prefecture aggregation	Lowest: Shiga 70.8; Yamanashi 74.7; Gifu 76.0; Niigata 76.1; Ibaraki 76.8	Prefecture aggregation
Prefecture‐clustered RIJ + ADI model	RIJ RR 0.919 (95% CI 0.894–0.945)	Prefecture‐clustered SEs
Suppressed cells imputed at 5 instead of 0	Outpatient Q5 81.5 (primary 81.3); inpatient Q5 108.8 (primary 108.8); outpatient RIJ RR 0.939 (0.922–0.956)	Midpoint imputation for suppressed SMA and national age–sex cells
Nearest‐three‐area Moran diagnostics	*I* = 0.084 outpatient; 0.406 inpatient; 0.238 suicide; 0.204 suicide‐model residual	Nearest‐three diagnostic

*Note*: Moran's *I* values are presented as descriptive diagnostics without formal permutation testing. Excluding the temporary COVID‐19 code I999 (31,445 claims, 0.038% of the outpatient source total) left Q1/Q5 outpatient SCRs (108.4/81.3) and the RIJ rate ratio (0.939, 95% CI 0.921–0.956) unchanged (Table S7).

Abbreviations: ADI, Area Deprivation Index; CI, confidence interval; RIJ, Rurality Index for Japan; SCR, standardized claim ratio; SMA, secondary medical area.

## DISCUSSION

### Principal findings

This nationwide ecological study identified a rurality‐related psychiatric SCR profile across Japan's secondary medical areas. An outpatient SCR below 100 indicates an area where locally billed outpatient volume is below the national age–sex benchmark and may warrant local review. Because the numerator reflects institution location while the denominator reflects resident population, a sub‐100 value may arise from cross‐boundary care rather than under‐provision. On this metric, more rural areas had lower outpatient psychiatric specialty therapy SCRs. Inpatient SCR was higher in the most rural than the most urban quintile, but the pattern was non‐monotonic, peaked in Q2, and continuous RIJ showed little correlation with inpatient SCR. Suicide SMR also rose across rurality quintiles, but the rurality–suicide association was largely attenuated after adjustment for deprivation. Together these findings indicate that rurality, claims‐based psychiatric service volume, socioeconomic deprivation, and residence‐based suicide mortality are related but distinct planning dimensions.

**Figure 1:**
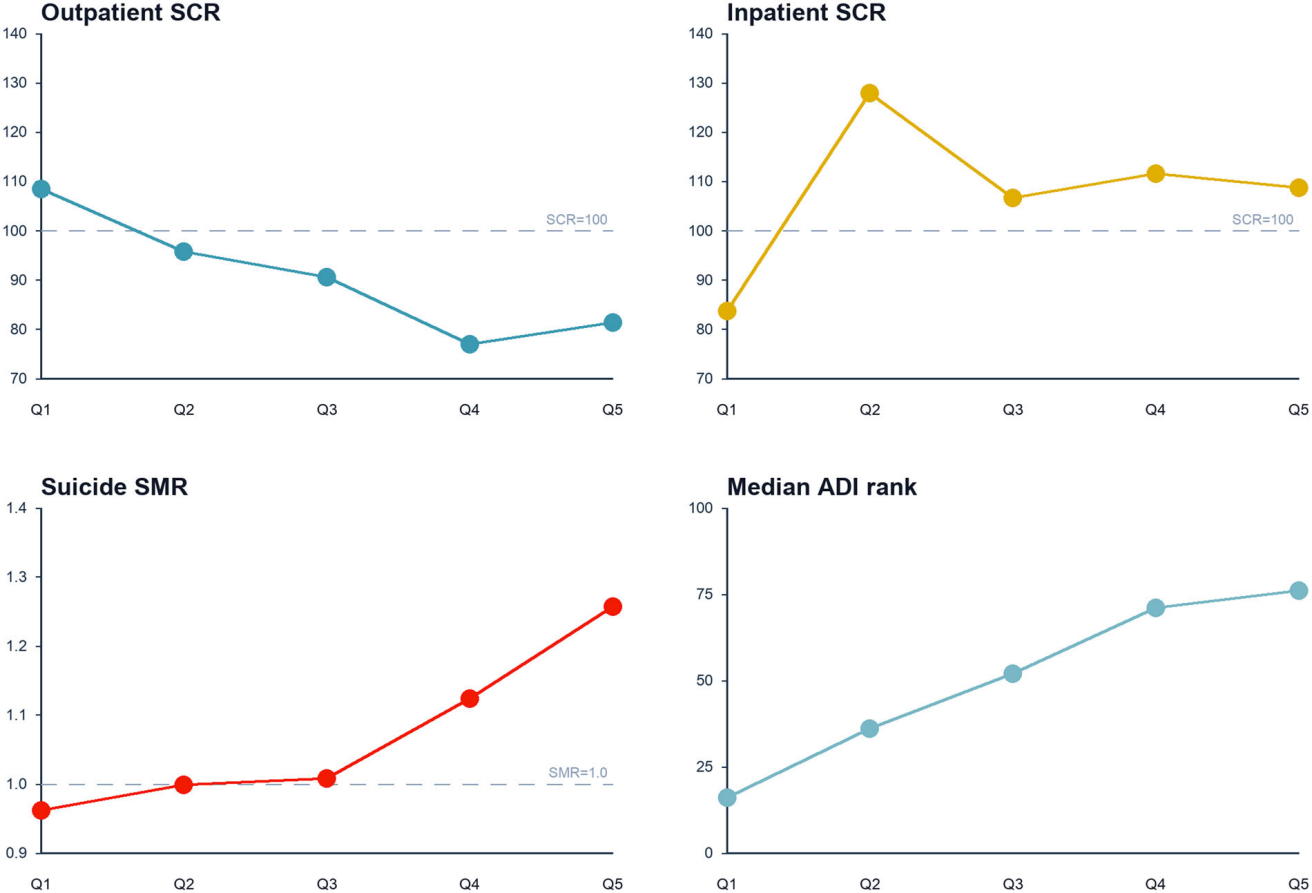
Psychiatric standardized claim ratio by institution location, suicide standardized mortality ratio, and deprivation by rurality. Values are summarized across 67 secondary medical areas in each Rurality Index for Japan quintile, ordered from Q1 (most urban) to Q5 (most rural). Outpatient and inpatient standardized claim ratios (SCRs) are group‐level observed claim counts divided by age–sex expected claim counts and multiplied by 100. Suicide standardized mortality ratio was calculated by indirect age–sex standardization. Area Deprivation Index rank is the median secondary‐medical‐area rank within each quintile. Dashed horizontal lines indicate the reference value of 100 for SCR and 1.0 for standardized mortality ratios. ADI, Area Deprivation Index; SMR, standardized mortality ratio.

The overlap analysis supports a screening‐indicator interpretation. Areas with low outpatient SCR, high inpatient SCR, and high suicide SMR were more common in Q4 and Q5, but such areas were not confined to the most rural quintile, and the two‐condition pattern of low outpatient and high inpatient SCR appeared across the rural–urban continuum. These combinations identify areas where local contextual review may be warranted rather than ranking regional performance. Because a low SCR does not by itself establish unmet need at the resident level, the cut‐point counts and Table 2 rate ratios are screening summaries, not causal estimates (Figure 1).

Because these counts dichotomize continuous ratios at single thresholds, areas near a cut‐point should not be over‐interpreted; continuous Spearman correlations and quasi‐Poisson models remain the main threshold‐free summaries.

### Interpretation in relation to prior work

The findings fit Japan's mental health service context, in which prior work has emphasized inpatient psychiatry, regional bed variation, and the need to strengthen community‐based services.^1^, ^2^, ^3^, ^4^ The annual 630 survey provides facility and patient stock information at a reference date, whereas SCR adds an annual claims‐flow benchmark of whether institutions in a policy unit generate psychiatric specialty therapy claims above or below age–sex expected volume.^5^ Recent NDB Open Data studies have described selected psychiatric service categories^16^, ^17^, ^18^; the present study extends that work by applying Japan's SCR planning logic to psychiatric specialty therapy claims within secondary medical areas and RIJ.^35^, ^39^ The higher inpatient SCR in Q5 than Q1 suggests a different service‐mix profile. However, the non‐monotonic pattern and Q2 peak do not support a simple rural gradient. A plausible interpretation, consistent with clinical experience in regional areas, is that psychiatric care in some rural areas still centers on inpatient treatment at hospitals with historically large bed capacities; inpatient claims may also reflect inter‐area admissions, length of stay, institutional volume, and coding rather than substitution for outpatient care. Because the facility measures used here are location proxies without bed‐capacity or workforce information, this hypothesis could not be tested directly.

The suicide SMR results add a contextual population‐outcome layer rather than a service‐to‐suicide causal pathway. Suicide mortality is shaped by social, economic, demographic, clinical, cultural, and reporting factors. Here, suicide SMR increased from 0.961 in the most urban to 1.257 in the most rural quintile, but the RIJ–suicide association attenuated from a rate ratio of 1.030–1.007 after including ADI rank, while ADI remained positively associated with suicide deaths relative to age–sex expected deaths. Rather than causal mediation, this attenuation reflects substantial overlap between the rurality gradient in suicide mortality and census‐based deprivation. This is consistent with Japanese ecological evidence that rurality and deprivation are related to mortality and suicide patterns.^19^, ^20^, ^21^, ^22^ It does not show that low outpatient claim volume caused higher suicide mortality, or that high suicide SMR indicates inadequate psychiatric care.

The findings also are consistent with the broader literature that access is multidimensional and should not be reduced to provider counts alone.^40^, ^41^, ^42^ Rural access measurement is sensitive to catchment size, travel behavior, and boundary crossing.^43^, ^44^ Here, the earlier adult‐population comparator and primary SCR produced similar outpatient contrasts, but nearest‐area pooling raised the Q5 median outpatient SCR from 62.2 to 98.1–101.1 across two to four pooled nearest areas, attenuating the rural–urban contrast. Moran diagnostics showed spatial clustering for inpatient SCR and suicide SMR but weaker evidence for outpatient SCR; these were treated as cautions because the analysis did not use a validated patient‐flow matrix. More broadly, as more health systems publish administrative claims by provider location, such open‐data indicators capture where care is billed rather than where residents live, and may misclassify cross‐boundary catchments as local shortfalls unless triangulated with residence‐based data.

### Policy and research implications

For psychiatry and service planning, psychiatric SCR, suicide SMR, and ADI are best used as complementary screening indicators requiring validation rather than automatic decision rules. SCR reframes the question from how many facilities or beds exist to whether local claim production is consistent with age–sex expected claim volume. In practice, prefectural planners and public health centers could: flag areas combining low outpatient SCR with high suicide SMR or high deprivation for local diagnostic review; compare outpatient and inpatient SCR patterns where service mix warrants closer examination; select areas where residence‐based claims, referral‐flow, workforce, bed‐capacity, travel‐time, and waiting‐time data should be obtained next; and track SCR annually as a descriptive monitoring signal rather than as a performance target or proof of under‐provision. This screening use is consistent with Japan's medical care planning emphasis on indicator‐based policy cycles.^35^, ^39^, ^45^

A related issue is whether deprivation indicates places where workforce supply is difficult despite apparent facility availability: OECD analyses have documented geographic imbalances in doctor supply, and the inverse care law holds that care availability may be least adequate where need is greatest.^46^, ^47^ In this study, however, ADI was not associated with lower total psychiatric facility density at the secondary‐medical‐area scale; instead, higher ADI was associated with lower clinic density and higher hospital density. This pattern is compatible with a hospital‐skewed provider configuration in more deprived areas, but it cannot establish whether deprived localities within those areas face workforce recruitment or retention problems. The secondary‐medical‐area scale may average over smaller inner‐city deprivation pockets, and facility counts are location proxies rather than capacity measures.

The next step is triangulation rather than direct allocation. Areas with low outpatient SCR should be examined together with qualitative knowledge from providers and service users, and areas with high suicide SMR require similarly broad review covering deprivation, isolation, employment, housing, welfare access, and suicide‐prevention systems. These indicators should not be used alone to allocate psychiatrists, label a region as definitively under‐provided, or judge the appropriateness of inpatient care.

For research, three extensions are important. First, patient‐residence‐based claims or controlled NDB analyses would distinguish low local service volume from cross‐boundary care, including whether residents receive treatment within or outside their own prefecture; this distinction may be particularly relevant for conditions such as developmental disorders and addictions, for which patients often travel to specialized urban hospitals. Second, psychiatric workforce data at the secondary‐medical‐area level, ideally full‐time‐equivalent and specialty‐specific, would help separate workforce capacity from service intensity. Third, epidemiology‐informed expected need should incorporate disorder‐specific prevalence, severity, age structure, help‐seeking, and care pathways rather than assuming uniform regional prevalence.

### Strengths and limitations

This study combined a validated rurality measure, national claims open data, official population denominators, municipal suicide statistics, a census‐based deprivation index, policy‐relevant secondary medical areas, and facility–location proxies across Japan. It separated institution–location claim volume from residence‐based suicide mortality and treated the analysis as explicitly ecological. Using the SCR framework aligned the service‐volume metric with an established Japanese health‐planning approach.

This study has several important limitations. First, NDB service outcomes are based on institution location, whereas suicide mortality is based on residence. Second, the ecological design is subject to the ecological fallacy, the modifiable areal unit problem, and unobserved patient flows; because the public NDB tables provide no residence‐to‐provider origin‐destination information, within‐prefecture and cross‐prefecture treatment could not be distinguished. Third, SCR standardizes against national age–sex claim rates, capturing age–sex expected claim volume rather than epidemiologic need; it does not incorporate prevalence, severity, help‐seeking, preferences, or clinical need. Fourth, claim counts reflect reimbursement categories, coding, and service intensity, not visit counts, quality, continuity, or individual trajectories. Fifth, NDB Open Data suppresses some small cells; suppressed cells were treated as zero before expected counts were anchored to national observed totals, and midpoint imputation changed the Q5 outpatient group‐level and median SCRs by approximately 0.2 points each (Tables S3 and S7). Sixth, large quasi‐Poisson dispersion indicated substantial residual between‐area heterogeneity, so estimates should be read as descriptive trends rather than precise causal, predictive, or mechanistic parameters.

Seventh, the overlap analysis dichotomized continuous indicators at fixed a priori cut‐points, so areas just below and above a threshold were counted as categorically different despite negligible differences; these counts are coarse screening summaries, not precise classifications. Threshold‐sensitivity analyses found that the qualitative concentration of the three‐condition pattern in Q4 and Q5 persisted, but near‐threshold bands still included 31 areas for outpatient SCR, 32 for inpatient SCR, and 84 for suicide SMR (Table S8).

Several measurement limitations should also be noted. Suicide counts may be unstable in small rural areas despite 3‐year pooling, and suicide SMR used broad age bands and a fixed 2024 population denominator. RIJ is not psychiatric‐access specific. ADI is ecological and 2020‐based, so it may not fully represent deprivation during 2023–2025. Facility counts capture reported specialties and locations, not psychiatrist FTE, consultation hours, or service capacity. Nearest‐area pooling and Moran diagnostics used geographic representatives rather than validated patient‐flow or adjacency structures; the number of pooled areas was a pragmatic choice, and island areas can be pooled with geographically distant areas, so attenuation under pooling indicates boundary sensitivity rather than specific patient flows.

## CONCLUSIONS

More rural Japanese secondary medical areas showed lower outpatient psychiatric specialty claim volume, billed by local institutions, relative to national age–sex benchmarks, while inpatient SCR was higher in the most rural than the most urban quintile but non‐monotonic and peaked in Q2. Suicide SMR was also higher in more rural and more deprived areas, but the rurality–suicide association was attenuated after adjustment for deprivation. The rural–urban contrast narrowed substantially when each area was pooled with its nearest areas, suggesting boundary sensitivity that may reflect cross‐boundary care and small‐area instability; these ecological findings are not stand‐alone estimates of epidemiologic unmet need, resident‐level access, service adequacy, causal effects, or required workforce. Psychiatric SCR, suicide SMR, and ADI may serve as candidate screening indicators for local psychiatric service assessment when triangulated with residence‐based claims, patient‐flow, workforce, travel, and local contextual data.

## AUTHOR CONTRIBUTIONS

Masahide Koda and Nahoko Harada conceptualized the study. Masahide Koda assembled the data, conducted the analyses, and drafted the manuscript. Shuhei Nomura, Makoto Kaneko, and Nahoko Harada contributed to interpretation and critically revised the manuscript. All authors approved the final manuscript.

## CONFLICT OF INTEREST STATEMENT

The authors declare no conflicts of interest.

## ETHICS APPROVAL STATEMENT

This study used aggregate administrative statistics and did not include individual‐level identifiable information. Formal ethical approval was not required for this aggregate‐data analysis under the applicable institutional policy.

## PATIENT CONSENT STATEMENT

Not applicable. This study used aggregate administrative statistics without individual‐level identifiable information.

## CLINICAL TRIAL REGISTRATION

Not applicable. This study is not a clinical trial.

## DECLARATION OF GENERATIVE AI AND AI‐ASSISTED TECHNOLOGIES

GPT‐5.5 Pro was used only for readability and language editing. The authors reviewed and approved the final manuscript and take full responsibility for its content.

## Supporting information

Supporting File 1

## Data Availability

Data are available from public open‐access repositories and from the corresponding author where redistribution is permitted. NDB Open Data, Medical Information Net Open Data, Basic Resident Register population data, National Land Numerical Information medical‐area boundary data, and the MHLW Basic Data on Suicide in Communities are publicly available from the sources cited in the References. Municipality‐level RIJ 2024 scores were obtained from the RIJ developers and should be used in accordance with source access conditions. The municipal Area Deprivation Index was calculated from publicly available census data following previously published methods.
