# Supplementary material for: Psychiatric standardized claim ratios by rurality and deprivation in Japan: A nationwide ecological study with contextual suicide mortality
Source: PCN Rep. 2026 Aug 10;5(3):e70392. doi: 10.1002/pcn5.70392 (PMC13455119; doi:10.1002/pcn5.70392)
Supplement: Supplementary file 1 [file PCN5-5-e70392-s001.docx]

# Supporting Information

Psychiatric standardized claim ratios by rurality and deprivation in Japan: a nationwide ecological study with contextual suicide mortality

Koda M, Nomura S, Kaneko M, Harada N.

## Contents

| **Item** | **Description** |
| --- | --- |
| Table S1 | NDB claim-item definitions and analysis roles |
| Table S2 | RIJ 2024 (mRIJ-direct) variant specification and observed score ranges |
| Table S3 | NDB source masking summary and SCR expected-count rescaling |
| Table S4 | Facility-density Spearman correlations |
| Table S5 | Age ranges and denominators by indicator |
| Table S6 | Quasi-Poisson RIJ quintile-categorical models |
| Table S7 | Sensitivity analyses: suppressed-cell midpoint imputation and COVID-19 temporary-code (I999) exclusion |
| Table S8 | Threshold sensitivity of overlap analysis |
| Table S9 | Nearest-area pooling sensitivity |

All tables are referenced in the main text. Tables S3, S7, S8, and S9 contain multiple panels (S3a-S3b; S7a-S7e; S8a-S8c; S9a-S9b).

## Table S1. NDB claim-item definitions and analysis roles

Psychiatric specialty therapy items in the NDB Open Data (fiscal year April 2023–March 2024), classified by their role in the analysis. “Primary” items contribute to the outpatient or inpatient claim-count outcomes; add-on settings were excluded. Counts below are numbers of unique fee-code rows. An item-level list of the 184 primary fee-code rows (with the 55 excluded add-on rows summarized) appears at the end of this document (Table S1, full list).

### Table S1a. Item counts by analysis role

| **Analysis role** | **NDB source setting** | **Fee-code rows, n** |
| --- | --- | --- |
| Primary outpatient claim-count outcome | Outpatient | 145 |
| Excluded outpatient add-on setting | Outpatient (add-on) | 49 |
| Primary inpatient contrast outcome | Inpatient | 39 |
| Excluded inpatient add-on setting | Inpatient (add-on) | 6 |

### Table S1b. Distinct claim categories (category code level)

Each row is a distinct billing category; a category may map to several fee-code rows. Setting indicates the NDB source setting (inpatient or outpatient). Category codes (e.g. I001, I002) are the official Japanese fee-schedule codes and serve as the linkage key to the NDB Open Data.

| **Setting** | **Code** | **Category name** | **Analysis role** |
| --- | --- | --- | --- |
| Inpatient | I000 | Electroconvulsive therapy | Primary inpatient contrast outcome |
| Inpatient | I000-2 | Transcranial magnetic stimulation | Primary inpatient contrast outcome |
| Inpatient | I001 | Inpatient psychotherapy | Primary inpatient contrast outcome |
| Inpatient | I002 | Outpatient/home-based psychotherapy | Primary inpatient contrast outcome |
| Inpatient | I002-3 | Continued psychiatric support fee for emergency patients | Primary inpatient contrast outcome |
| Inpatient | I003 | Standard psychoanalytic therapy | Primary inpatient contrast outcome |
| Inpatient | I004 | Psychosomatic medicine therapy | Primary inpatient contrast outcome |
| Inpatient | I005 | Inpatient group psychotherapy | Primary inpatient contrast outcome |
| Inpatient | I007 | Psychiatric occupational therapy | Primary inpatient contrast outcome |
| Inpatient | I008 | Inpatient life-skills training therapy | Primary inpatient contrast outcome |
| Inpatient | I008-2 | Psychiatric short-stay care | Primary inpatient contrast outcome |
| Inpatient | I009 | Psychiatric day care | Primary inpatient contrast outcome |
| Inpatient | I011 | Psychiatric discharge guidance fee | Primary inpatient contrast outcome |
| Inpatient | I011-2 | Pre-discharge home-visit guidance fee | Primary inpatient contrast outcome |
| Inpatient | I012-2 | Psychiatric home-nursing instruction fee | Primary inpatient contrast outcome |
| Inpatient | I013 | Antipsychotic specific-drug treatment management fee | Primary inpatient contrast outcome |
| Inpatient | I014 | Involuntary-admission care fee | Primary inpatient contrast outcome |
| Inpatient (add-on) | General/note add-on items |  | Excluded inpatient add-on setting |
| Outpatient | I000 | Electroconvulsive therapy | Primary outpatient claim-count outcome |
| Outpatient | I000-2 | Transcranial magnetic stimulation | Primary outpatient claim-count outcome |
| Outpatient | I001 | Inpatient psychotherapy | Primary outpatient claim-count outcome |
| Outpatient | I002 | Outpatient/home-based psychotherapy | Primary outpatient claim-count outcome |
| Outpatient | I002-2 | Continued outpatient support and guidance fee | Primary outpatient claim-count outcome |
| Outpatient | I002-3 | Continued psychiatric support fee for emergency patients | Primary outpatient claim-count outcome |
| Outpatient | I003 | Standard psychoanalytic therapy | Primary outpatient claim-count outcome |
| Outpatient | I003-2 | Cognitive/cognitive-behavioral therapy | Primary outpatient claim-count outcome |
| Outpatient | I004 | Psychosomatic medicine therapy | Primary outpatient claim-count outcome |
| Outpatient | I006 | Outpatient group psychotherapy | Primary outpatient claim-count outcome |
| Outpatient | I006-2 | Addiction group therapy | Primary outpatient claim-count outcome |
| Outpatient | I007 | Psychiatric occupational therapy | Primary outpatient claim-count outcome |
| Outpatient | I008-2 | Psychiatric short-stay care | Primary outpatient claim-count outcome |
| Outpatient | I009 | Psychiatric day care | Primary outpatient claim-count outcome |
| Outpatient | I010 | Psychiatric night care | Primary outpatient claim-count outcome |
| Outpatient | I010-2 | Psychiatric day-and-night care | Primary outpatient claim-count outcome |
| Outpatient | I012 | Psychiatric home-nursing and guidance fee | Primary outpatient claim-count outcome |
| Outpatient | I012-2 | Psychiatric home-nursing instruction fee | Primary outpatient claim-count outcome |
| Outpatient | I013 | Antipsychotic specific-drug treatment management fee | Primary outpatient claim-count outcome |
| Outpatient | I014 | Involuntary-admission care fee | Primary outpatient claim-count outcome |
| Outpatient | I015 | Severe-dementia patient day-care fee | Primary outpatient claim-count outcome |
| Outpatient | I016 | Psychiatric home-patient support management fee | Primary outpatient claim-count outcome |
| Outpatient | I999 | Care for mental disorders (temporary COVID-19 code) | Primary outpatient claim-count outcome |
| Outpatient (add-on) | General/note add-on items |  | Excluded outpatient add-on setting |

## Table S2. RIJ 2024 (mRIJ-direct) variant specification

Specification of the Rurality Index for Japan (RIJ) 2024 variant used, its components, secondary-medical-area (SMA) aggregation method, observed score ranges, and quintile cut-points (Q1 most urban to Q5 most rural).

| **Item** | **Value** |
| --- | --- |
| RIJ 2024 variant used | mRIJ-direct |
| Source score | Municipality-level RIJ 2024 score (rurality-scale sheet for ordinary municipalities, RIJ 2024 dataset) |
| Distance component | Straight-line distance to nearest secondary or tertiary emergency hospital |
| Island component | Modified island coding: no emergency hospital on island=1, island with emergency hospital=0.5, otherwise=0 |
| Other components | Population density and special heavy-snowfall area indicator |
| SMA aggregation | Population-weighted mean of municipality-level mRIJ-direct scores |
| SMA-level score range | 2.876-97.985 |
| Municipality-level mRIJ-direct observed range | 1-100 (n=1,897) |
| Q1 most urban score range within quintile | 2.876-36.000 |
| Q2 score range within quintile | 36.275-47.501 |
| Q3 score range within quintile | 47.502-57.513 |
| Q4 score range within quintile | 57.591-65.899 |
| Q5 most rural score range within quintile | 66.000-97.985 |

### Table S2b. Observed municipality-level mRIJ-direct range

| **Item** | **Value** |
| --- | --- |
| Municipality-level mRIJ-direct observed n | 1897 |
| Municipality-level mRIJ-direct observed min | 1 |
| Municipality-level mRIJ-direct observed max | 100 |
| Municipality-level mRIJ-direct observed median | 58 |

## Table S3. NDB masking and SCR expected-count rescaling

### Table S3a. NDB source masking summary

NDB Open Data suppress cells with fewer than 10 claims. Suppressed cells were treated as zero in the primary analysis. Percentages are of total SMA cells per outcome.

| **Outcome** | **Setting** | **Fee-code rows** | **SMA cells** | **Suppressed cells** | **Suppressed, %** | **Displayed SMA total** | **Source national total** |
| --- | --- | --- | --- | --- | --- | --- | --- |
| Outpatient claims excluding add-on | Outpatient | 145 | 48575 | 8111 | 16.7 | 81717926 | 81869246 |
| Inpatient claims excluding add-on | Inpatient | 39 | 13065 | 2404 | 18.4 | 45997763 | 46032888 |

### Table S3b. SCR expected-count rescaling

Expected counts were rescaled so that the national expected total equalled the national observed displayed-SMA total. Claim counts are shown to one decimal; rescale factors are shown to six decimals for reproducibility.

| **Outcome** | **National observed (SMA)** | **Sum expected (raw)** | **Sum expected (rescaled)** | **Obs − rescaled exp** | **Rescale factor** |
| --- | --- | --- | --- | --- | --- |
| outpatient_claims_non_addition | 81717926 | 81857679.9 | 81717926 | 0 | 0.998293 |
| inpatient_claims_non_addition | 45997763 | 46030088.9 | 45997763 | -7.450580596923828e-9 | 0.999298 |

Formula. Expected counts were first computed as sum(area age-sex population x national age-sex claim rate); raw expected counts were then multiplied by the rescale factor so national expected equalled national observed displayed SMA claims.

## Table S4. Facility-density Spearman correlations

Spearman rank correlations (rho) between facility-density measures and RIJ, ADI rank, outpatient SCR, inpatient SCR, and suicide SMR across 335 secondary medical areas.

| **Facility-density measure** | **Comparator** | **n** | **Spearman rho** |
| --- | --- | --- | --- |
| Psychiatric facilities per 100,000 | RIJ | 335 | -0.028 |
| Psychiatric facilities per 100,000 | ADI rank | 335 | 0.004 |
| Psychiatric facilities per 100,000 | Outpatient SCR | 335 | 0.316 |
| Psychiatric facilities per 100,000 | Inpatient SCR | 335 | 0.2 |
| Psychiatric facilities per 100,000 | Suicide SMR | 335 | 0.022 |
| Psychiatric clinics per 100,000 | RIJ | 335 | -0.353 |
| Psychiatric clinics per 100,000 | ADI rank | 335 | -0.338 |
| Psychiatric clinics per 100,000 | Outpatient SCR | 335 | 0.338 |
| Psychiatric clinics per 100,000 | Inpatient SCR | 335 | -0.041 |
| Psychiatric clinics per 100,000 | Suicide SMR | 335 | -0.161 |
| Psychiatric hospitals per 100,000 | RIJ | 335 | 0.455 |
| Psychiatric hospitals per 100,000 | ADI rank | 335 | 0.458 |
| Psychiatric hospitals per 100,000 | Outpatient SCR | 335 | 0.054 |
| Psychiatric hospitals per 100,000 | Inpatient SCR | 335 | 0.432 |
| Psychiatric hospitals per 100,000 | Suicide SMR | 335 | 0.219 |

## Table S5. Age ranges and denominators by indicator

Numerator source, age range, and denominator/expected-count construction for each indicator.

| **Indicator** | **Numerator** | **Age range** | **Denominator / expected count** |
| --- | --- | --- | --- |
| Outpatient/inpatient psychiatric claim-count SCR | NDB Open Data facility-location claim counts, April 2023-March 2024 | All ages in 5-year bands: 0-4, 5-9, ..., 85-89, 90+ | 2024 Basic Resident Register age-sex population x national age-sex claim rates |
| Descriptive population total | Not applicable | Residents aged 20 years or older | 2024 Basic Resident Register adult population, used only for descriptive population summaries and adult-population comparator diagnostics |
| Suicide SMR | MHLW Basic Data on Suicide in Communities, A7 resident-based deaths, April 2022-March 2025 | Sex-specific A7 age bands, aggregated to secondary medical areas | Area age-sex population x national age-sex suicide rates over the same 36-month period |
| Area Deprivation Index | Not applicable | Census socioeconomic indicators, not an individual-level age-restricted outcome | Municipality-level ADI 2020 aggregated to secondary medical areas by population weighting |

## Table S6. Quasi-Poisson RIJ quintile-categorical models

Rate ratios (95% confidence intervals) from quasi-Poisson offset models with RIJ quintile as a categorical predictor (Q1 reference), with and without ADI rank. Dispersion phi shown. n = 335 secondary medical areas; all models converged.

| **Outcome** | **Model** | **Term** | **Rate ratio (95% CI)** | **Dispersion phi** |
| --- | --- | --- | --- | --- |
| Outpatient psychiatric specialty therapy claims | RIJ quintile only | Q2 vs Q1 | 0.883 (0.805-0.968) | 27357.3 |
| Outpatient psychiatric specialty therapy claims | RIJ quintile only | Q3 vs Q1 | 0.835 (0.744-0.938) | 27357.3 |
| Outpatient psychiatric specialty therapy claims | RIJ quintile only | Q4 vs Q1 | 0.709 (0.607-0.829) | 27357.3 |
| Outpatient psychiatric specialty therapy claims | RIJ quintile only | Q5 vs Q1 | 0.750 (0.613-0.918) | 27357.3 |
| Outpatient psychiatric specialty therapy claims | RIJ quintile + ADI rank | Q2 vs Q1 | 0.862 (0.781-0.951) | 27449.9 |
| Outpatient psychiatric specialty therapy claims | RIJ quintile + ADI rank | Q3 vs Q1 | 0.804 (0.707-0.915) | 27449.9 |
| Outpatient psychiatric specialty therapy claims | RIJ quintile + ADI rank | Q4 vs Q1 | 0.670 (0.562-0.800) | 27449.9 |
| Outpatient psychiatric specialty therapy claims | RIJ quintile + ADI rank | Q5 vs Q1 | 0.701 (0.560-0.879) | 27449.9 |
| Outpatient psychiatric specialty therapy claims | RIJ quintile + ADI rank | ADI rank per 10 points | 1.014 (0.994-1.034) | 27449.9 |
| Inpatient psychiatric specialty therapy claims | RIJ quintile only | Q2 vs Q1 | 1.528 (1.298-1.800) | 53868.1 |
| Inpatient psychiatric specialty therapy claims | RIJ quintile only | Q3 vs Q1 | 1.275 (1.040-1.564) | 53868.1 |
| Inpatient psychiatric specialty therapy claims | RIJ quintile only | Q4 vs Q1 | 1.333 (1.050-1.693) | 53868.1 |
| Inpatient psychiatric specialty therapy claims | RIJ quintile only | Q5 vs Q1 | 1.300 (0.956-1.769) | 53868.1 |
| Inpatient psychiatric specialty therapy claims | RIJ quintile + ADI rank | Q2 vs Q1 | 1.268 (1.079-1.491) | 45722.6 |
| Inpatient psychiatric specialty therapy claims | RIJ quintile + ADI rank | Q3 vs Q1 | 0.935 (0.755-1.157) | 45722.6 |
| Inpatient psychiatric specialty therapy claims | RIJ quintile + ADI rank | Q4 vs Q1 | 0.850 (0.655-1.104) | 45722.6 |
| Inpatient psychiatric specialty therapy claims | RIJ quintile + ADI rank | Q5 vs Q1 | 0.770 (0.555-1.068) | 45722.6 |
| Inpatient psychiatric specialty therapy claims | RIJ quintile + ADI rank | ADI rank per 10 points | 1.109 (1.075-1.145) | 45722.6 |
| Suicide deaths | RIJ quintile only | Q2 vs Q1 | 1.039 (1.004-1.075) | 2.9 |
| Suicide deaths | RIJ quintile only | Q3 vs Q1 | 1.048 (1.006-1.093) | 2.9 |
| Suicide deaths | RIJ quintile only | Q4 vs Q1 | 1.169 (1.113-1.227) | 2.9 |
| Suicide deaths | RIJ quintile only | Q5 vs Q1 | 1.307 (1.230-1.390) | 2.9 |
| Suicide deaths | RIJ quintile + ADI rank | Q2 vs Q1 | 0.993 (0.960-1.027) | 2.4 |
| Suicide deaths | RIJ quintile + ADI rank | Q3 vs Q1 | 0.976 (0.935-1.018) | 2.4 |
| Suicide deaths | RIJ quintile + ADI rank | Q4 vs Q1 | 1.049 (0.995-1.105) | 2.4 |
| Suicide deaths | RIJ quintile + ADI rank | Q5 vs Q1 | 1.150 (1.078-1.227) | 2.4 |
| Suicide deaths | RIJ quintile + ADI rank | ADI rank per 10 points | 1.026 (1.020-1.033) | 2.4 |

## Table S7. Sensitivity analyses

Two sensitivity analyses are reported: (i) imputing suppressed source cells at the midpoint value of 5 instead of 0, with national age-sex claim rates, expected counts, and rescaling factors recalculated (S7a, S7b, S7e); and (ii) excluding the temporary COVID-19 reimbursement code I999 from the primary outpatient outcome (S7c, S7d).

### Table S7a. Group-level and area-level SCR under midpoint imputation

SCR by RIJ quintile with suppressed cells imputed at 5, alongside the primary (zero-imputation) values and the difference. Differences are small (≤0.3 points), indicating minimal distortion from the suppression rule.

| **Outcome** | **RIJ quintile** | **Observed** | **Expected** | **SCR (group)** | **SCR (median)** | **Primary SCR (group)** | **Primary SCR (median)** | **Δ group** | **Δ median** |
| --- | --- | --- | --- | --- | --- | --- | --- | --- | --- |
| Outpatient claims | Q1 most urban | 67 | 48489436 | 108.4 | 93.9 | 108.4 | 93.9 | -0.03 | -0.03 |
| Outpatient claims | Q2 | 67 | 16478527 | 95.7 | 95.4 | 95.7 | 95.4 | 0 | -0.01 |
| Outpatient claims | Q3 | 67 | 9291494 | 90.6 | 85.4 | 90.6 | 85.4 | 0.03 | 0.03 |
| Outpatient claims | Q4 | 67 | 4782050 | 77 | 73 | 76.9 | 72.9 | 0.08 | 0.06 |
| Outpatient claims | Q5 most rural | 67 | 2716974 | 81.5 | 62.4 | 81.3 | 62.2 | 0.17 | 0.22 |
| Inpatient claims | Q1 most urban | 67 | 19694657 | 83.6 | 75.3 | 83.6 | 75.3 | -0.01 | -0.01 |
| Inpatient claims | Q2 | 67 | 12778896 | 127.8 | 116.8 | 127.8 | 116.8 | -0.01 | -0.01 |
| Inpatient claims | Q3 | 67 | 6622417 | 106.7 | 92.9 | 106.7 | 92.9 | 0.01 | 0 |
| Inpatient claims | Q4 | 67 | 4452846 | 111.6 | 112 | 111.5 | 112 | 0.03 | -0.02 |
| Inpatient claims | Q5 most rural | 67 | 2460967 | 108.8 | 100 | 108.8 | 99.8 | 0.07 | 0.21 |

### Table S7b. Offset models under midpoint imputation (outpatient)

| **Outcome** | **Sensitivity** | **Model** | **Term** | **Rate ratio (95% CI)** | **Dispersion phi** |
| --- | --- | --- | --- | --- | --- |
| Outpatient claims | Suppressed cells imputed at 5 | RIJ only | RIJ per 10 points | 0.939 (0.922-0.956) | 25927.2 |
| Outpatient claims | Suppressed cells imputed at 5 | RIJ + ADI rank | RIJ per 10 points | 0.919 (0.897-0.942) | 25632.4 |
| Outpatient claims | Suppressed cells imputed at 5 | RIJ + ADI rank | ADI rank per 10 points | 1.026 (1.006-1.047) | 25632.4 |

### Table S7c. COVID-19 temporary-code (I999) exclusion summary

| **Metric** | **Value** | **Denominator** | **Percent** |
| --- | --- | --- | --- |
| I999 national source claims | 31445 | 81869246 | 0.038 |
| I999 displayed SMA claims | 30213 | 81717926 | 0.037 |

Outpatient model and rescaling values after excluding code I999:

| **Quantity (I999 excluded)** | **Value** |
| --- | --- |
| RIJ-only RR per 10 points | 0.939 |
| RIJ-only 95% CI lower | 0.921 |
| RIJ-only 95% CI upper | 0.956 |
| dispersion phi | 25923 |
| expected rescale factor | 0.998 |

### Table S7d. Outpatient SCR by RIJ quintile, excluding I999

| **RIJ quintile** | **SMAs** | **Observed** | **Expected** | **SCR (group)** | **SCR (median)** |
| --- | --- | --- | --- | --- | --- |
| Q1 most urban | 67 | 48460433 | 44695684 | 108.4 | 93.9 |
| Q2 | 67 | 16464801 | 17206263 | 95.7 | 95.4 |
| Q3 | 67 | 9280298 | 10248047 | 90.6 | 85.3 |
| Q4 | 67 | 4772861 | 6206456 | 76.9 | 73 |
| Q5 most rural | 67 | 2709320 | 3331264 | 81.3 | 62.2 |

### Table S7e. Reproducibility QC for midpoint imputation

Full-precision quality-control values for the midpoint-imputation recomputation (observed totals, raw and rescaled expected totals, rescale factors, and cell counts).

| **Setting** | **Imputed value** | **Observed total** | **Raw expected total** | **Rescale factor** | **Rescaled expected total** | **Age-sex claim total** | **Age-sex suppr. cells** | **SMA suppr. cells** | **SMA source cells** |
| --- | --- | --- | --- | --- | --- | --- | --- | --- | --- |
| Outpatient | 5 | 81758481 | 81864669.4 | 1.00 | 81758481.0 | 81870466 | 1398 | 8111 | 48575 |
| Inpatient | 5 | 46009783 | 46032563.7 | 1.00 | 46009783.0 | 46034015 | 495 | 2404 | 13065 |

## Table S8. Threshold sensitivity of overlap analysis

Overlap-count sensitivity analyses varied the outpatient SCR, inpatient SCR, and suicide SMR cut-points.

## Table S8a. Summary of one-at-a-time threshold sensitivity

| Condition | Cut-point changes | Total count range | Q4+Q5 share range |
| --- | --- | --- | --- |
| 2 conditions | 10 one-at-a-time rows | 70-114; median 96 | 48.2-58.3%; median 50.3% |
| 3 conditions | 15 one-at-a-time rows | 40-83; median 61 | 54.2-77.5%; median 63.6% |

## Table S8b. Full threshold-grid summary

| Condition | Cut-point combinations | Total count range | Q4+Q5 share range |
| --- | --- | --- | --- |
| 2 conditions | 25 | 62-134; median 95 | 46.3-58.3%; median 50.0% |
| 3 conditions | 125 | 24-113; median 56 | 49.3-81.6%; median 63.9% |

## Table S8c. Near-threshold bands

| Indicator | Band | Total | Q1 | Q2 | Q3 | Q4 | Q5 |
| --- | --- | --- | --- | --- | --- | --- | --- |
| Outpatient SCR | 95-105 | 31 | 9 | 10 | 6 | 4 | 2 |
| Inpatient SCR | 95-105 | 32 | 4 | 7 | 7 | 4 | 10 |
| Suicide SMR | 0.95-1.05 | 84 | 22 | 18 | 19 | 15 | 10 |

## Table S9. Nearest-area pooling sensitivity

Sensitivity of the nearest-area pooling analysis to the number of pooled geographically nearest areas (k) and to island focal areas. Pools were formed from approximate geographic representatives (bounding-box centers) of secondary-medical-area boundary polygons; observed and expected outpatient claims were summed within each pool before recalculating SCR. The pooling is a boundary-sensitivity (catchment-smoothing) diagnostic and does not use a validated adjacency or patient-flow structure.

### Table S9a. Q5 median pooled outpatient SCR by number of pooled nearest areas

| Nearest areas pooled, k | Areas per pool | Q5 median pooled outpatient SCR | Median of maximum within-pool distance in Q5, km |
| --- | --- | --- | --- |
| 1 | 2 | 80.0 | 36.1 |
| 2 | 3 | 98.1 | 42.5 |
| 3 (main analysis) | 4 | 101.1 | 49.6 |
| 4 | 5 | 99.8 | 55.7 |
| 5 | 6 | 101.9 | 61.6 |
| 6 | 7 | 101.0 | 68.9 |
| 10 | 11 | 98.9 | 91.2 |

### Table S9b. Island focal areas and nearest-three-area pooling (Q5)

| Focal areas | n | Median focal outpatient SCR | Median nearest-three-area pooled SCR |
| --- | --- | --- | --- |
| All Q5 areas | 67 | 62.2 | 101.1 |
| Q5 excluding island focal areas | 54 | 65.3 | 96.4 |
| Q5 island focal areas only | 13 | 53.9 | 109.1 |

Note. Q5, most rural Rurality Index for Japan quintile; SCR, standardized claim ratio. The three-nearest-area pooling used in the main analysis was a pragmatic boundary-sensitivity choice; the attenuation of the low Q5 median outpatient SCR was similar with two or four pooled nearest areas. Under the representative-point approximation, island focal areas can be pooled with geographically distant areas (maximum within-pool distance up to 978 km for the Tokyo island area); pooled values should therefore be read as boundary-sensitivity diagnostics, not as estimates of specific patient flows.

## Table S1 (full list). NDB fee-code rows

Item-level listing of the 184 primary psychiatric specialty therapy fee-code rows used to construct the outpatient and inpatient claim-count outcomes, with NDB source setting, category code, service name, points, and analysis role. The 49 outpatient and 6 inpatient add-on fee-code rows that were excluded are summarized in the final two rows. Category codes (e.g. I001) are the official Japanese fee-schedule codes and serve as the linkage key to the NDB Open Data.

| **Setting** | **Code** | **Service name** | **Points** | **Analysis role** |
| --- | --- | --- | --- | --- |
| Inpatient | I000 | Electroconvulsive therapy (other than type 1) | 150 | Primary inpatient contrast outcome |
| Inpatient | I000-2 | Transcranial magnetic stimulation | 1200 | Primary inpatient contrast outcome |
| Inpatient | I001 | Inpatient psychotherapy (type 2; within 6 months of admission; per session) | 150 | Primary inpatient contrast outcome |
| Inpatient | I002 | Outpatient psychotherapy (first visit, ≥60 min, with initial consultation fee; 1; per session) | 540 | Primary inpatient contrast outcome |
| Inpatient | I002-3 | Continued psychiatric support fee for emergency patients (inpatient) | 900 | Primary inpatient contrast outcome |
| Inpatient | I003 | Standard psychoanalytic therapy (per session) | 390 | Primary inpatient contrast outcome |
| Inpatient | I004 | Psychosomatic medicine therapy (inpatient; per session) | 150 | Primary inpatient contrast outcome |
| Inpatient | I005 | Inpatient group psychotherapy (per day) | 100 | Primary inpatient contrast outcome |
| Inpatient | I007 | Psychiatric occupational therapy (per day) | 220 | Primary inpatient contrast outcome |
| Inpatient | I008 | Inpatient life-skills training therapy (beyond 6 months of admission) | 75 | Primary inpatient contrast outcome |
| Inpatient | I008-2 | Psychiatric short-stay care (small-scale; inpatient) | 138 | Primary inpatient contrast outcome |
| Inpatient | I009 | Psychiatric day care (small-scale; inpatient) | 295 | Primary inpatient contrast outcome |
| Inpatient | I011 | Psychiatric discharge guidance fee | 320 | Primary inpatient contrast outcome |
| Inpatient | I011-2 | Pre-discharge home-visit guidance fee | 380 | Primary inpatient contrast outcome |
| Inpatient | I012-2 | Psychiatric home-nursing instruction fee | 300 | Primary inpatient contrast outcome |
| Inpatient | I013 | Treatment-resistant schizophrenia management fee | 500 | Primary inpatient contrast outcome |
| Inpatient | I014 | Involuntary-admission care fee | 300 | Primary inpatient contrast outcome |
| Inpatient | I001 | Inpatient psychotherapy (type 2; beyond 6 months of admission; per session) | 80 | Primary inpatient contrast outcome |
| Inpatient | I001 | Inpatient psychotherapy (type 1; per session) | 400 | Primary inpatient contrast outcome |
| Inpatient | I008 | Inpatient life-skills training therapy (within 6 months of admission) | 100 | Primary inpatient contrast outcome |
| Inpatient | I000 | Electroconvulsive therapy (with closed-circuit general anesthesia (mask or intubation)) | 2800 | Primary inpatient contrast outcome |
| Inpatient | I001 | Family inpatient psychotherapy (type 1; per session) | 400 | Primary inpatient contrast outcome |
| Inpatient | I001 | Family inpatient psychotherapy (type 2; within 6 months of admission; per session) | 150 | Primary inpatient contrast outcome |
| Inpatient | I001 | Family inpatient psychotherapy (type 2; beyond 6 months of admission; per session) | 80 | Primary inpatient contrast outcome |
| Inpatient | I008-2 | Psychiatric short-stay care (large-scale; inpatient) | 165 | Primary inpatient contrast outcome |
| Inpatient | I008-2 | Psychiatric short-stay care (small-scale; early add-on, inpatient) | 148 | Primary inpatient contrast outcome |
| Inpatient | I008-2 | Psychiatric short-stay care (large-scale; early add-on, inpatient) | 175 | Primary inpatient contrast outcome |
| Inpatient | I009 | Psychiatric day care (large-scale; inpatient) | 350 | Primary inpatient contrast outcome |
| Inpatient | I009 | Psychiatric day care (small-scale; early, inpatient) | 320 | Primary inpatient contrast outcome |
| Inpatient | I009 | Psychiatric day care (large-scale; early, inpatient) | 375 | Primary inpatient contrast outcome |
| Inpatient | I009 | Psychiatric day care (large-scale; >3 years, >3 days/week; inpatient) | 315 | Primary inpatient contrast outcome |
| Inpatient | I013 | Long-acting injectable antipsychotic treatment management fee (inpatient) | 250 | Primary inpatient contrast outcome |
| Inpatient | I002 | Outpatient psychotherapy (other than (a) and (b); <30 min; other than type 1; per session) | 315 | Primary inpatient contrast outcome |
| Inpatient | I002 | Outpatient psychotherapy (other than (a) and (b); ≥30 min; other than type 1; per session) | 390 | Primary inpatient contrast outcome |
| Inpatient | I002 | Outpatient psychotherapy (first visit, ≥60 min, with initial consultation fee; by a designated mental-health physician; per session) | 560 | Primary inpatient contrast outcome |
| Inpatient | I002 | Outpatient psychotherapy (other than (a) and (b); ≥30 min; by a designated mental-health physician; per session) | 410 | Primary inpatient contrast outcome |
| Inpatient | I002 | Outpatient psychotherapy (other than (a) and (b); <30 min; by a designated mental-health physician; per session) | 330 | Primary inpatient contrast outcome |
| Inpatient | I002 | Family outpatient psychotherapy (<30 min; by a designated mental-health physician; per session) | 330 | Primary inpatient contrast outcome |
| Inpatient | I002 | Outpatient psychotherapy (≥3 antidepressants, MHLW criteria not met; <30 min; by a designated mental-health physician) | 165 | Primary inpatient contrast outcome |
| Outpatient | I000 | Electroconvulsive therapy (other than type 1) | 150 | Primary outpatient claim-count outcome |
| Outpatient | I000-2 | Transcranial magnetic stimulation | 1200 | Primary outpatient claim-count outcome |
| Outpatient | I001 | Inpatient psychotherapy (type 2; within 6 months of admission; per session) | 150 | Primary outpatient claim-count outcome |
| Outpatient | I002 | Outpatient psychotherapy (post-involuntary-discharge patient, by the care-plan psychiatrist during the planned support period) | 660 | Primary outpatient claim-count outcome |
| Outpatient | I002-2 | Continued outpatient support and guidance fee (per day) | 55 | Primary outpatient claim-count outcome |
| Outpatient | I002-3 | Continued psychiatric support fee for emergency patients (non-inpatient) | 300 | Primary outpatient claim-count outcome |
| Outpatient | I003 | Standard psychoanalytic therapy (per session) | 390 | Primary outpatient claim-count outcome |
| Outpatient | I003-2 | Cognitive/cognitive-behavioral therapy (physician and nurse jointly; per day) | 350 | Primary outpatient claim-count outcome |
| Outpatient | I004 | Psychosomatic medicine therapy (inpatient; per session) | 150 | Primary outpatient claim-count outcome |
| Outpatient | I006 | Outpatient group psychotherapy (per day) | 270 | Primary outpatient claim-count outcome |
| Outpatient | I006-2 | Addiction group therapy (drug dependence; per session) | 340 | Primary outpatient claim-count outcome |
| Outpatient | I007 | Psychiatric occupational therapy (per day) | 220 | Primary outpatient claim-count outcome |
| Outpatient | I008-2 | Psychiatric short-stay care (small-scale; per day) | 275 | Primary outpatient claim-count outcome |
| Outpatient | I009 | Psychiatric day care (small-scale; per day) | 590 | Primary outpatient claim-count outcome |
| Outpatient | I010 | Psychiatric night care (per day) | 540 | Primary outpatient claim-count outcome |
| Outpatient | I010-2 | Psychiatric day-and-night care (per day) | 1000 | Primary outpatient claim-count outcome |
| Outpatient | I012 | Psychiatric home-nursing and guidance fee (type 1; by public-health nurse or nurse, up to the 3rd day/week ≥30 min) | 580 | Primary outpatient claim-count outcome |
| Outpatient | I012-2 | Psychiatric home-nursing instruction fee | 300 | Primary outpatient claim-count outcome |
| Outpatient | I013 | Long-acting injectable antipsychotic treatment management fee (non-inpatient) | 250 | Primary outpatient claim-count outcome |
| Outpatient | I014 | Involuntary-admission care fee | 300 | Primary outpatient claim-count outcome |
| Outpatient | I015 | Severe-dementia patient day-care fee (per day) | 1040 | Primary outpatient claim-count outcome |
| Outpatient | I016 | Psychiatric home-patient support management fee (patients designated by MHLW needing intensive support, single-building, 1 patient; once monthly) | 3000 | Primary outpatient claim-count outcome |
| Outpatient | I999 | Care for mental disorders (temporary COVID-19 code) (temporary COVID-19 reimbursement handling) | 147 | Primary outpatient claim-count outcome |
| Outpatient | I009 | Psychiatric day care (large-scale; per day) | 700 | Primary outpatient claim-count outcome |
| Outpatient | I001 | Inpatient psychotherapy (type 2; beyond 6 months of admission; per session) | 80 | Primary outpatient claim-count outcome |
| Outpatient | I001 | Inpatient psychotherapy (type 1; per session) | 400 | Primary outpatient claim-count outcome |
| Outpatient | I000 | Electroconvulsive therapy (with closed-circuit general anesthesia (mask or intubation)) | 2800 | Primary outpatient claim-count outcome |
| Outpatient | I004 | Psychosomatic medicine therapy (outpatient; per session; follow-up visit) | 80 | Primary outpatient claim-count outcome |
| Outpatient | I004 | Psychosomatic medicine therapy (outpatient; per session; first visit) | 110 | Primary outpatient claim-count outcome |
| Outpatient | I008-2 | Psychiatric short-stay care (large-scale; per day) | 330 | Primary outpatient claim-count outcome |
| Outpatient | I012 | Psychiatric home-nursing and guidance fee (type 1; by public-health nurse or nurse, up to the 3rd day/week <30 min) | 445 | Primary outpatient claim-count outcome |
| Outpatient | I012 | Psychiatric home-nursing and guidance fee (type 1; by public-health nurse or nurse, from the 4th day/week ≥30 min) | 680 | Primary outpatient claim-count outcome |
| Outpatient | I012 | Psychiatric home-nursing and guidance fee (type 1; by public-health nurse or nurse, from the 4th day/week <30 min) | 530 | Primary outpatient claim-count outcome |
| Outpatient | I012 | Psychiatric home-nursing and guidance fee (type 1; by assistant nurse, up to the 3rd day/week ≥30 min) | 530 | Primary outpatient claim-count outcome |
| Outpatient | I012 | Psychiatric home-nursing and guidance fee (type 1; by assistant nurse, up to the 3rd day/week <30 min) | 405 | Primary outpatient claim-count outcome |
| Outpatient | I012 | Psychiatric home-nursing and guidance fee (type 1; by assistant nurse, from the 4th day/week ≥30 min) | 630 | Primary outpatient claim-count outcome |
| Outpatient | I012 | Psychiatric home-nursing and guidance fee (type 1; by assistant nurse, from the 4th day/week <30 min) | 490 | Primary outpatient claim-count outcome |
| Outpatient | I013 | Treatment-resistant schizophrenia management fee | 500 | Primary outpatient claim-count outcome |
| Outpatient | I008-2 | Psychiatric short-stay care (large-scale; inpatient) | 165 | Primary outpatient claim-count outcome |
| Outpatient | I009 | Psychiatric day care (large-scale; inpatient) | 350 | Primary outpatient claim-count outcome |
| Outpatient | I009 | Psychiatric day care (large-scale; early, inpatient) | 375 | Primary outpatient claim-count outcome |
| Outpatient | I012 | Psychiatric home-nursing and guidance fee (type 3; by public-health nurse or nurse, same day, 2 patients, up to the 3rd day/week ≥30 min) | 580 | Primary outpatient claim-count outcome |
| Outpatient | I012 | Psychiatric home-nursing and guidance fee (type 3; by public-health nurse or nurse, same day, 2 patients, up to the 3rd day/week <30 min) | 445 | Primary outpatient claim-count outcome |
| Outpatient | I012 | Psychiatric home-nursing and guidance fee (type 3; by public-health nurse or nurse, same day, 2 patients, from the 4th day/week ≥30 min) | 680 | Primary outpatient claim-count outcome |
| Outpatient | I012 | Psychiatric home-nursing and guidance fee (type 3; by public-health nurse or nurse, same day, 2 patients, from the 4th day/week <30 min) | 530 | Primary outpatient claim-count outcome |
| Outpatient | I012 | Psychiatric home-nursing and guidance fee (type 3; by public-health nurse or nurse, same day, ≥3 patients, up to the 3rd day/week ≥30 min) | 293 | Primary outpatient claim-count outcome |
| Outpatient | I012 | Psychiatric home-nursing and guidance fee (type 3; by public-health nurse or nurse, same day, ≥3 patients, up to the 3rd day/week <30 min) | 225 | Primary outpatient claim-count outcome |
| Outpatient | I012 | Psychiatric home-nursing and guidance fee (type 3; by public-health nurse or nurse, same day, ≥3 patients, from the 4th day/week ≥30 min) | 343 | Primary outpatient claim-count outcome |
| Outpatient | I012 | Psychiatric home-nursing and guidance fee (type 3; by public-health nurse or nurse, same day, ≥3 patients, from the 4th day/week <30 min) | 268 | Primary outpatient claim-count outcome |
| Outpatient | I012 | Psychiatric home-nursing and guidance fee (type 3; by assistant nurse, same day, 2 patients, up to the 3rd day/week ≥30 min) | 530 | Primary outpatient claim-count outcome |
| Outpatient | I012 | Psychiatric home-nursing and guidance fee (type 3; by assistant nurse, same day, 2 patients, up to the 3rd day/week <30 min) | 405 | Primary outpatient claim-count outcome |
| Outpatient | I012 | Psychiatric home-nursing and guidance fee (type 3; by assistant nurse, same day, 2 patients, from the 4th day/week ≥30 min) | 630 | Primary outpatient claim-count outcome |
| Outpatient | I012 | Psychiatric home-nursing and guidance fee (type 3; by assistant nurse, same day, 2 patients, from the 4th day/week <30 min) | 490 | Primary outpatient claim-count outcome |
| Outpatient | I012 | Psychiatric home-nursing and guidance fee (type 3; by assistant nurse, same day, ≥3 patients, up to the 3rd day/week ≥30 min) | 268 | Primary outpatient claim-count outcome |
| Outpatient | I012 | Psychiatric home-nursing and guidance fee (type 3; by assistant nurse, same day, ≥3 patients, up to the 3rd day/week <30 min) | 205 | Primary outpatient claim-count outcome |
| Outpatient | I012 | Psychiatric home-nursing and guidance fee (type 3; by assistant nurse, same day, ≥3 patients, from the 4th day/week ≥30 min) | 318 | Primary outpatient claim-count outcome |
| Outpatient | I012 | Psychiatric home-nursing and guidance fee (type 3; by assistant nurse, same day, ≥3 patients, from the 4th day/week <30 min) | 248 | Primary outpatient claim-count outcome |
| Outpatient | I009 | Psychiatric day care (small-scale; >3 years, >3 days/week; per day) | 531 | Primary outpatient claim-count outcome |
| Outpatient | I009 | Psychiatric day care (large-scale; >3 years, >3 days/week; per day) | 630 | Primary outpatient claim-count outcome |
| Outpatient | I010 | Psychiatric night care (>3 years, >3 days/week; per day) | 486 | Primary outpatient claim-count outcome |
| Outpatient | I010-2 | Psychiatric day-and-night care (>3 years, >3 days/week; per day) | 900 | Primary outpatient claim-count outcome |
| Outpatient | I009 | Psychiatric day care (small-scale; >3 years, >3 days/week; inpatient) | 266 | Primary outpatient claim-count outcome |
| Outpatient | I002-2 | Continued outpatient support and guidance fee (≥3 antidepressants, MHLW criteria not met; excluding note 2; per day) | 28 | Primary outpatient claim-count outcome |
| Outpatient | I002 | Home-based psychotherapy (post-involuntary-discharge patient, by the care-plan psychiatrist during the planned support period) | 660 | Primary outpatient claim-count outcome |
| Outpatient | I002 | Outpatient psychotherapy (post-involuntary-discharge patient; care-plan psychiatrist; ≥3 antidepressants, MHLW criteria not met) | 330 | Primary outpatient claim-count outcome |
| Outpatient | I002 | Home-based psychotherapy (post-involuntary-discharge patient; care-plan psychiatrist; ≥3 antidepressants, MHLW criteria not met) | 330 | Primary outpatient claim-count outcome |
| Outpatient | I003-2 | Cognitive/cognitive-behavioral therapy (by physician; per day) | 480 | Primary outpatient claim-count outcome |
| Outpatient | I016 | Psychiatric home-patient support management fee (patients designated by MHLW, single-building, 1 patient; once monthly) | 2500 | Primary outpatient claim-count outcome |
| Outpatient | I016 | Psychiatric home-patient support management fee (patients designated by MHLW, single-building, ≥2 patients; once monthly) | 1875 | Primary outpatient claim-count outcome |
| Outpatient | I016 | Psychiatric home-patient support management fee (patients designated by MHLW, single-building, 1 patient; once monthly) | 2056 | Primary outpatient claim-count outcome |
| Outpatient | I016 | Psychiatric home-patient support management fee (patients designated by MHLW, single-building, ≥2 patients; once monthly) | 1542 | Primary outpatient claim-count outcome |
| Outpatient | I002 | Outpatient psychotherapy (post-involuntary-discharge patient; care-plan psychiatrist; ≥3 antidepressants, criteria not met; post-discharge continued-support add-on) | 468 | Primary outpatient claim-count outcome |
| Outpatient | I006-2 | Addiction group therapy (gambling dependence; per session) | 300 | Primary outpatient claim-count outcome |
| Outpatient | I012 | Psychiatric home-nursing and guidance fee (type 1; by occupational therapist, up to the 3rd day/week ≥30 min) | 580 | Primary outpatient claim-count outcome |
| Outpatient | I012 | Psychiatric home-nursing and guidance fee (type 1; by occupational therapist, up to the 3rd day/week <30 min) | 445 | Primary outpatient claim-count outcome |
| Outpatient | I012 | Psychiatric home-nursing and guidance fee (type 1; by occupational therapist, from the 4th day/week ≥30 min) | 680 | Primary outpatient claim-count outcome |
| Outpatient | I012 | Psychiatric home-nursing and guidance fee (type 1; by occupational therapist, from the 4th day/week <30 min) | 530 | Primary outpatient claim-count outcome |
| Outpatient | I012 | Psychiatric home-nursing and guidance fee (type 1; by psychiatric social worker, up to the 3rd day/week ≥30 min) | 580 | Primary outpatient claim-count outcome |
| Outpatient | I012 | Psychiatric home-nursing and guidance fee (type 1; by psychiatric social worker, up to the 3rd day/week <30 min) | 445 | Primary outpatient claim-count outcome |
| Outpatient | I012 | Psychiatric home-nursing and guidance fee (type 1; by psychiatric social worker, from the 4th day/week ≥30 min) | 680 | Primary outpatient claim-count outcome |
| Outpatient | I012 | Psychiatric home-nursing and guidance fee (type 1; by psychiatric social worker, from the 4th day/week <30 min) | 530 | Primary outpatient claim-count outcome |
| Outpatient | I012 | Psychiatric home-nursing and guidance fee (type 3; by occupational therapist, same day, 2 patients, up to the 3rd day/week ≥30 min) | 580 | Primary outpatient claim-count outcome |
| Outpatient | I012 | Psychiatric home-nursing and guidance fee (type 3; by occupational therapist, same day, 2 patients, up to the 3rd day/week <30 min) | 445 | Primary outpatient claim-count outcome |
| Outpatient | I012 | Psychiatric home-nursing and guidance fee (type 3; by occupational therapist, same day, 2 patients, from the 4th day/week ≥30 min) | 680 | Primary outpatient claim-count outcome |
| Outpatient | I012 | Psychiatric home-nursing and guidance fee (type 3; by occupational therapist, same day, 2 patients, from the 4th day/week <30 min) | 530 | Primary outpatient claim-count outcome |
| Outpatient | I012 | Psychiatric home-nursing and guidance fee (type 3; by occupational therapist, same day, ≥3 patients, up to the 3rd day/week ≥30 min) | 293 | Primary outpatient claim-count outcome |
| Outpatient | I012 | Psychiatric home-nursing and guidance fee (type 3; by occupational therapist, same day, ≥3 patients, up to the 3rd day/week <30 min) | 225 | Primary outpatient claim-count outcome |
| Outpatient | I012 | Psychiatric home-nursing and guidance fee (type 3; by occupational therapist, same day, ≥3 patients, from the 4th day/week ≥30 min) | 343 | Primary outpatient claim-count outcome |
| Outpatient | I012 | Psychiatric home-nursing and guidance fee (type 3; by occupational therapist, same day, ≥3 patients, from the 4th day/week <30 min) | 268 | Primary outpatient claim-count outcome |
| Outpatient | I012 | Psychiatric home-nursing and guidance fee (type 3; by psychiatric social worker, same day, 2 patients, up to the 3rd day/week ≥30 min) | 580 | Primary outpatient claim-count outcome |
| Outpatient | I012 | Psychiatric home-nursing and guidance fee (type 3; by psychiatric social worker, same day, 2 patients, up to the 3rd day/week <30 min) | 445 | Primary outpatient claim-count outcome |
| Outpatient | I012 | Psychiatric home-nursing and guidance fee (type 3; by psychiatric social worker, same day, 2 patients, from the 4th day/week ≥30 min) | 680 | Primary outpatient claim-count outcome |
| Outpatient | I012 | Psychiatric home-nursing and guidance fee (type 3; by psychiatric social worker, same day, 2 patients, from the 4th day/week <30 min) | 530 | Primary outpatient claim-count outcome |
| Outpatient | I012 | Psychiatric home-nursing and guidance fee (type 3; by psychiatric social worker, same day, ≥3 patients, up to the 3rd day/week ≥30 min) | 293 | Primary outpatient claim-count outcome |
| Outpatient | I012 | Psychiatric home-nursing and guidance fee (type 3; by psychiatric social worker, same day, ≥3 patients, up to the 3rd day/week <30 min) | 225 | Primary outpatient claim-count outcome |
| Outpatient | I012 | Psychiatric home-nursing and guidance fee (type 3; by psychiatric social worker, same day, ≥3 patients, from the 4th day/week ≥30 min) | 343 | Primary outpatient claim-count outcome |
| Outpatient | I012 | Psychiatric home-nursing and guidance fee (type 3; by psychiatric social worker, same day, ≥3 patients, from the 4th day/week <30 min) | 268 | Primary outpatient claim-count outcome |
| Outpatient | I016 | Psychiatric home-patient support management fee (single-building, 1 patient; once monthly) | 2030 | Primary outpatient claim-count outcome |
| Outpatient | I016 | Psychiatric home-patient support management fee (single-building, ≥2 patients; once monthly) | 1248 | Primary outpatient claim-count outcome |
| Outpatient | I002 | Outpatient psychotherapy (first visit, ≥60 min, with initial consultation fee; 1; per session) | 540 | Primary outpatient claim-count outcome |
| Outpatient | I002 | Outpatient psychotherapy (first visit ≥60 min; ≥3 antidepressants, MHLW criteria not met; 1) | 270 | Primary outpatient claim-count outcome |
| Outpatient | I002 | Outpatient psychotherapy (other than (a) and (b); <30 min; other than type 1; per session) | 315 | Primary outpatient claim-count outcome |
| Outpatient | I002 | Outpatient psychotherapy (other than (a) and (b); ≥30 min; other than type 1; per session) | 390 | Primary outpatient claim-count outcome |
| Outpatient | I002 | Outpatient psychotherapy (≥3 antidepressants, MHLW criteria not met; <30 min; other than type 1) | 158 | Primary outpatient claim-count outcome |
| Outpatient | I002 | Outpatient psychotherapy (≥3 antidepressants, MHLW criteria not met; ≥30 min; other than type 1) | 195 | Primary outpatient claim-count outcome |
| Outpatient | I002 | Home-based psychotherapy (other than (a) and (b); 30–60 min; other than type 1; per session) | 390 | Primary outpatient claim-count outcome |
| Outpatient | I002 | Home-based psychotherapy (first visit, ≥60 min, with initial consultation fee; 1; per session) | 600 | Primary outpatient claim-count outcome |
| Outpatient | I002 | Home-based psychotherapy (first visit ≥60 min; ≥3 antidepressants, MHLW criteria not met; 1) | 300 | Primary outpatient claim-count outcome |
| Outpatient | I002 | Home-based psychotherapy (other than (a) and (b); ≥60 min; other than type 1; per session) | 530 | Primary outpatient claim-count outcome |
| Outpatient | I002 | Home-based psychotherapy (other than (a) and (b); <30 min; other than type 1; per session) | 315 | Primary outpatient claim-count outcome |
| Outpatient | I002 | Home-based psychotherapy (≥3 antidepressants, MHLW criteria not met; <30 min; other than type 1) | 158 | Primary outpatient claim-count outcome |
| Outpatient | I002 | Home-based psychotherapy (≥3 antidepressants, MHLW criteria not met; 30–60 min; other than type 1) | 195 | Primary outpatient claim-count outcome |
| Outpatient | I002 | Family outpatient psychotherapy (≥3 antidepressants, MHLW criteria not met; <30 min; other than type 1) | 158 | Primary outpatient claim-count outcome |
| Outpatient | I002 | Family outpatient psychotherapy (≥3 antidepressants, MHLW criteria not met; ≥30 min; other than type 1) | 195 | Primary outpatient claim-count outcome |
| Outpatient | I002 | Family outpatient psychotherapy (<30 min; other than type 1; per session) | 315 | Primary outpatient claim-count outcome |
| Outpatient | I002 | Family outpatient psychotherapy (≥30 min; other than type 1; per session) | 390 | Primary outpatient claim-count outcome |
| Outpatient | I002 | Family home-based psychotherapy (≥60 min; other than type 1; per session) | 530 | Primary outpatient claim-count outcome |
| Outpatient | I002 | Family home-based psychotherapy (≥3 antidepressants, MHLW criteria not met; <30 min; other than type 1) | 158 | Primary outpatient claim-count outcome |
| Outpatient | I002 | Family home-based psychotherapy (<30 min; other than type 1; per session) | 315 | Primary outpatient claim-count outcome |
| Outpatient | I002 | Family home-based psychotherapy (30–60 min; other than type 1; per session) | 390 | Primary outpatient claim-count outcome |
| Outpatient | I006-2 | Addiction group therapy (alcohol dependence; per session) | 300 | Primary outpatient claim-count outcome |
| Outpatient | I002 | Outpatient psychotherapy (first visit, ≥60 min, with initial consultation fee; by a designated mental-health physician; per session) | 560 | Primary outpatient claim-count outcome |
| Outpatient | I002 | Outpatient psychotherapy (other than (a) and (b); ≥30 min; by a designated mental-health physician; per session) | 410 | Primary outpatient claim-count outcome |
| Outpatient | I002 | Outpatient psychotherapy (other than (a) and (b); <30 min; by a designated mental-health physician; per session) | 330 | Primary outpatient claim-count outcome |
| Outpatient | I002 | Home-based psychotherapy (first visit, ≥60 min, with initial consultation fee; by a designated mental-health physician; per session) | 620 | Primary outpatient claim-count outcome |
| Outpatient | I002 | Home-based psychotherapy (other than (a) and (b); ≥60 min; by a designated mental-health physician; per session) | 550 | Primary outpatient claim-count outcome |
| Outpatient | I002 | Home-based psychotherapy (other than (a) and (b); 30–60 min; by a designated mental-health physician; per session) | 410 | Primary outpatient claim-count outcome |
| Outpatient | I002 | Home-based psychotherapy (other than (a) and (b); <30 min; by a designated mental-health physician; per session) | 330 | Primary outpatient claim-count outcome |
| Outpatient | I002 | Family outpatient psychotherapy (≥30 min; by a designated mental-health physician; per session) | 410 | Primary outpatient claim-count outcome |
| Outpatient | I002 | Family outpatient psychotherapy (<30 min; by a designated mental-health physician; per session) | 330 | Primary outpatient claim-count outcome |
| Outpatient | I002 | Family home-based psychotherapy (≥60 min; by a designated mental-health physician; per session) | 550 | Primary outpatient claim-count outcome |
| Outpatient | I002 | Family home-based psychotherapy (30–60 min; by a designated mental-health physician; per session) | 410 | Primary outpatient claim-count outcome |
| Outpatient | I002 | Family home-based psychotherapy (<30 min; by a designated mental-health physician; per session) | 330 | Primary outpatient claim-count outcome |
| Outpatient | I002 | Outpatient psychotherapy (first visit ≥60 min; ≥3 antidepressants, MHLW criteria not met; by a designated mental-health physician) | 280 | Primary outpatient claim-count outcome |
| Outpatient | I002 | Outpatient psychotherapy (≥3 antidepressants, MHLW criteria not met; ≥30 min; by a designated mental-health physician) | 205 | Primary outpatient claim-count outcome |
| Outpatient | I002 | Outpatient psychotherapy (≥3 antidepressants, MHLW criteria not met; <30 min; by a designated mental-health physician) | 165 | Primary outpatient claim-count outcome |
| Outpatient | I002 | Home-based psychotherapy (first visit ≥60 min; ≥3 antidepressants, MHLW criteria not met; by a designated mental-health physician) | 310 | Primary outpatient claim-count outcome |
| Outpatient | I002 | Home-based psychotherapy (≥3 antidepressants, MHLW criteria not met; 30–60 min; by a designated mental-health physician) | 205 | Primary outpatient claim-count outcome |
| Outpatient | I002 | Home-based psychotherapy (≥3 antidepressants, MHLW criteria not met; <30 min; by a designated mental-health physician) | 165 | Primary outpatient claim-count outcome |
| Outpatient | I002 | Family outpatient psychotherapy (≥3 antidepressants, MHLW criteria not met; ≥30 min; by a designated mental-health physician) | 205 | Primary outpatient claim-count outcome |
| Outpatient | I002 | Family outpatient psychotherapy (≥3 antidepressants, MHLW criteria not met; <30 min; by a designated mental-health physician) | 165 | Primary outpatient claim-count outcome |
| Outpatient | I002 | Family home-based psychotherapy (≥3 antidepressants, MHLW criteria not met; 30–60 min; designated mental-health physician) | 205 | Primary outpatient claim-count outcome |
| Outpatient | I002 | Family home-based psychotherapy (≥3 antidepressants, MHLW criteria not met; <30 min; by a designated mental-health physician) | 165 | Primary outpatient claim-count outcome |
| Outpatient | I002 | Home-based psychotherapy (≥3 antidepressants, MHLW criteria not met; ≥60 min; by a designated mental-health physician) | 275 | Primary outpatient claim-count outcome |
| Outpatient | I002 | Home-based psychotherapy (≥3 antidepressants, MHLW criteria not met; ≥60 min; other than type 1) | 265 | Primary outpatient claim-count outcome |
| Outpatient (add-on) |  | Excluded outpatient add-on items (49 fee-code rows) |  | Excluded outpatient add-on setting |
| Inpatient (add-on) |  | Excluded inpatient add-on items (6 fee-code rows) |  | Excluded inpatient add-on setting |
